# Surface Temperature Assisted State of Charge Estimation for Retired Power Batteries

**DOI:** 10.3390/s25154863

**Published:** 2025-08-07

**Authors:** Liangyu Xu, Wenxuan Han, Jiawei Dong, Ke Chen, Yuchen Li, Guangchao Geng

**Affiliations:** 1 College of Electrical Engineering, Zhejiang University, Hangzhou 310027, China; 3220103135@zju.edu.cn (L.X.); hanwx@zju.edu.cn (W.H.); 3200100547@zju.edu.cn (J.D.); 3220102756@zju.edu.cn (K.C.); 3220104860@zju.edu.cn (Y.L.); 2 International Research Center for Advanced Electrical Engineering, Zhejiang University, Haining 314499, China

**Keywords:** retired power batteries, state of charge estimation, surface temperature characteristics, gated recurrent unit, thermal feature extraction

## Abstract

Accurate State of Charge (SOC) estimation for retired power batteries remains a critical challenge due to their degraded electrochemical properties and heterogeneous aging mechanisms. Traditional methods relying solely on electrical parameters (e.g., voltage and current) exhibit significant errors, as aged batteries experience altered internal resistance, capacity fade, and uneven heat generation, which distort the relationship between electrical signals and actual SOC. To address these limitations, this study proposes a surface temperature-assisted SOC estimation method, leveraging the distinct thermal characteristics of retired batteries. By employing infrared thermal imaging, key temperature feature regions—the positive/negative tabs and central area—are identified, which exhibit strong correlations with SOC dynamics under varying operational conditions. A Gated Recurrent Unit (GRU) neural network is developed to integrate multi-region temperature data with electrical parameters, capturing spatial–temporal thermal–electrical interactions unique to retired batteries. The model is trained and validated using experimental data collected under constant current discharge conditions, demonstrating superior accuracy compared to conventional methods. Specifically, our method achieves 64.3–68.1% lower RMSE than traditional electrical-parameter-only approaches (V-I inputs) across 0.5 C–2 C discharge rates. Results show that the proposed method reduces SOC estimation errors compared to traditional voltage-based models, achieving RMSE values below 1.04 across all tested rates. This improvement stems from the model’s ability to decode localized heating patterns and their hysteresis effects, which are particularly pronounced in aged batteries. The method’s robustness under high-rate operations highlights its potential for enhancing the reliability of retired battery management systems in secondary applications such as energy storage.

## 1. Introduction

The rapid development of electric vehicles (EVs) has become a crucial strategy for addressing climate change and achieving carbon neutrality goals [1]. With global EV sales reaching 10 million units in 2022 [2], the automotive industry faces an impending challenge of managing retired power batteries. These retired batteries typically retain 70–80% of their initial capacity [3], making them valuable for secondary applications such as energy storage systems and low-speed electric vehicles [4]. However, the effective utilization of retired batteries requires accurate state of charge (SOC) estimation, which remains a significant technical challenge due to battery aging and performance degradation [5].

Traditional SOC estimation methods primarily rely on electrical parameters including voltage, current, and internal resistance [6]. The ampere-hour integration method, while simple to implement, suffers from cumulative errors that become particularly pronounced in retired batteries due to capacity fade [7]. Model-based approaches such as Kalman filters require precise battery models that are difficult to establish for aged batteries with heterogeneous material degradation [8]. Recent studies by Solomon et al. [9] revealed that conventional electrical parameter-based methods exhibit up to 35% higher SOC estimation errors when applied to retired batteries compared to fresh ones.

The fundamental challenge lies in the complex aging mechanisms of lithium-ion batteries. As demonstrated by Broussely et al. [10], retired batteries experience multiple degradation modes including lithium inventory loss, active material dissolution, and solid electrolyte interface (SEI) layer growth. These aging processes significantly alter the relationship between electrical parameters and actual SOC, rendering traditional estimation methods ineffective [11]. Moreover, the increased heterogeneity in electrode materials leads to uneven current distribution and localized heating effects that cannot be captured by conventional measurement techniques. This heterogeneity manifests as spatially variable thermal signatures during charge/discharge cycles, which correlate strongly with SOC dynamics in degraded cells [12].

The distinct thermal behavior of retired batteries provides a unique opportunity to overcome electrical-based limitations. Unlike fresh batteries, retired cells exhibit pronounced thermal inhomogeneity due to three aging-specific phenomena:

1. Material degradation: SEI growth and lithium plating increase polarization resistance, elevating irreversible Joule heating. Bandhauer et al. [13] confirmed that irreversible heat contributes > 60% to total heat generation in aged LiFePO_4_ batteries—significantly higher than in fresh cells (<40%).
2. Spatial thermal variance: Heterogeneous electrode degradation (e.g., graphite fragmentation, active material dissolution) creates localized hotspots correlated with SOC-dependent electrochemical reactions. Richardson et al. [14] demonstrated that tab and central regions display distinct thermal hysteresis patterns during discharge, especially at 30–70% SOC.
3. Enhanced thermal–electrical decoupling: Temperature fluctuations in retired batteries better reflect SOC-dependent entropic changes than voltage/current signals [15]. Chen et al. [16] validated that surface temperature variations reduce SOC errors by 18% in aged batteries when fused with voltage data, confirming thermal parameters’ compensatory role for electrical signal degradation.

Despite this potential, existing thermal-assisted methods face three critical limitations:

1. Spatial resolution: Single-point sensors (e.g., thermocouples) fail to capture regional thermal hysteresis. Xiao [17] reported measurement errors exceeding 2 C when using contact sensors on curved surfaces, misrepresenting thermal gradients.
2. Measurement latency: Conventional thermistors introduce delays (>2 s) that misalign thermal and electrical data streams during dynamic operations [18].
3. Quantitative modeling: No studies establish multi-region thermal-SOC correlations for retired batteries [19], particularly under high-rate conditions where thermal effects dominate.

Infrared (IR) thermal imaging addresses these gaps by enabling high-resolution (0.1 C) real-time surface temperature mapping [20]. Recent advances by Wang et al. [21] demonstrated that IR imaging detects localized overheating in aged batteries with spatial resolution unattainable by contact sensors. Sequino and Vaglieco [22] further established that IR-derived temperature differentials between tabs and central regions correlate with SOC hysteresis in degraded Li-ion cells. Building on this, our preliminary experiments identified three thermally responsive zones—the positive tab, negative tab, and central region—that exhibit SOC-dependent hysteresis patterns unique to retired batteries.

This study bridges the identified research gaps through three key contributions:

1. Infrared thermal feature extraction: Identification of optimal surface temperature feature regions through spatial–temporal analysis of IR data, overcoming the resolution limitations of point-based sensors.
2. BiGRU-based multi-modal fusion: Development of a bidirectional gated recurrent unit neural network architecture integrating temporal electrical parameters with spatial thermal characteristics, specifically designed to capture degradation-induced hysteresis patterns through bidirectional sequence learning.
3. Degradation-adaptive modeling: Experimental validation under variable current conditions (0.5 C–2 C) demonstrating robustness against aging heterogeneity—a critical advance for second-life applications.

The remainder of this paper is organized as follows: Section 2 details the infrared thermal imaging analysis and temperature feature region selection. Section 3 describes the GRU-based SOC estimation model development. Section 4 presents experimental results and comparative analysis. Section 5 concludes with the main findings and future research directions.

## 2. Acquisition of Surface Temperature Feature Regions of Retired Power Battery

### 2.1. Principle of Infrared Thermal Imaging and Thermal Feature Region Identification

The working principle of infrared thermal imaging is based on capturing the infrared radiation emitted by objects. All objects with temperatures above absolute zero (−273.15 °C) will emit infrared rays. The infrared thermal imager uses special sensors to detect these invisible rays and converts them into visible thermal images through computer processing. For retired power batteries, this technology can show the real-time temperature distribution on the battery surface without touching the battery itself, which is very safe and convenient.

In normal lithium-ion batteries, the heat generation mainly comes from two parts. The first part is called “irreversible heat”, which is like the heat generated when we rub our hands together. This kind of heat is caused by the current passing through the internal resistance of the battery. The second part is called “reversible heat”, which is related to the chemical reactions inside the battery. When lithium ions move between the positive and negative electrodes, they will absorb or release heat. These two types of heat together determine the temperature changes on the battery surface.

However, the situation becomes different in retired power batteries. After long-term use, the internal materials of the battery will age. For example, the graphite material on the negative electrode will gradually break and fall off, just like pencil lead being worn out. This aging causes the battery’s internal resistance to increase, leading to substantially elevated irreversible heat generation during operation compared to new batteries. Calorimetric measurements confirm that such aging effects can increase the irreversible heat contribution by over 50% in aged LiFePO_4_ cells. This is why retired batteries become hotter during use compared to new batteries.

The infrared thermal imager can clearly show these temperature differences. When we look at the thermal images, we can see that the hottest spots usually appear near the metal tabs (the positive and negative terminals). This is because these areas have the highest current density. Imagine many cars passing through a narrow tunnel—the entrance and exit will be the busiest. Similarly, the current is most concentrated near the tabs when entering and exiting the battery.

Another important discovery is that the temperature of the central area changes in a special pattern during discharge. In the early stage of discharge, the central area is cooler than the tabs. But as the discharge continues, the central temperature gradually catches up and even surpasses the tab temperature. This phenomenon can be explained by the “heat conduction delay”. The heat generated at the tabs needs time to spread to the center, just like when we pour hot water into a thermos, the outer wall gets hot first and the inner temperature rises later.

Infrared thermal imaging reveals that the temperature differential (ΔT) between tabs and the central region exhibits strong hysteresis during discharge, with peak sensitivity at 30–70% SOC where entropy effects dominate Joule heating [23]. This characteristic pattern provides important support for our selection of temperature feature regions.

It is worth noting that the thermal characteristics of retired batteries are more pronounced than those of new batteries. Comparative experiments show that aged cells exhibit significantly higher surface temperature heterogeneity due to inhomogeneous current distribution and material degradation [24]. This thermal amplification effect makes infrared thermal imaging particularly suitable for SOC estimation of retired batteries.

### 2.2. Experimental Platform Setup and Procedure

**Figure 1:**
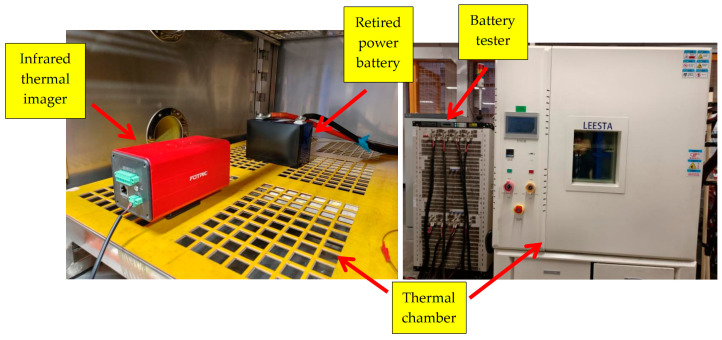
Experimental operation platform. The FOTRIC-626C thermal imager (thermal sensitivity < 0.05 °C @30 °C) was mounted inside the thermal chamber, targeting the frontal surface of the retired prismatic battery where both cathode (top-left) and anode (top-right) terminals are visible.

The experimental platform included a retired prismatic battery (Rept Battero Energy Co., Ltd., Wenzhou, China, REPU Limited, Dongfeng E70 model, parameters in Table 1), a CE-600 battery tester, a FOTRIC-626C thermal imager, a thermal chamber, and a host computer. The CE-600 tester supports constant current/voltage and variable-current modes (0.05% accuracy), providing synchronized electrical parameter recordings for GRU model training. The thermal chamber maintained an ambient temperature at 25 °C to eliminate environmental interference. The experimental platform is shown in Figure 1.

Experimental steps:

1. Place the battery in the thermal chamber and connect it to the battery tester.
2. Execute variable-current charging/discharging (0.5 C-rate) while recording electrical parameters.
3. Align the thermal imager to capture surface thermal videos.
4. Export temperature matrices and electrical data for analysis.

### 2.3. Analysis and Selection of Temperature Feature Regions

By analyzing infrared thermal images at different SOC, significant dynamic surface temperature characteristics of retired power batteries can be observed. During the constant current-constant voltage (CC-CV) charging phase (0% → 100% SOC), the temperature of the positive electrode tab continuously increases, while the temperature rise in the central region is relatively smaller. This phenomenon occurs because lithium ions diffuse from the positive tab towards the center during constant current charging, leading to higher current density near the tab and local accumulation of ohmic heat. In the constant current discharging phase (100% → 0% SOC), the temperature of the positive tab rises rapidly in the early stage of discharge; when the SOC drops to 50%, the temperature in the central region begins to exceed that of the tab; by the end of discharge, the central region reaches its peak temperature, while the tab temperature slightly decreases due to the current cutoff. The experiments covered the full SOC window (0% → 100%), defined as the usable capacity range under controlled discharge conditions. Table 2 shows the infrared thermal images of the battery at different charging and discharging stages.

These observations can be explained by the thermal conduction mechanism. In the early stage of discharge, heat concentrated at the positive tab is conducted towards the center through the aluminum foil current collector. However, due to the high current, the heat generation rate exceeds the dissipation rate, keeping the tab as the high-temperature zone. As the discharge progresses, the central region becomes the primary heat source as it continuously receives conducted heat and its own polarization resistance increases. By analyzing the spatial–temporal evolution of the temperature field during the entire charge–discharge process, the ‘positive tab, negative tab, and central region’ are identified as key surface temperature feature regions for characterizing SOC changes.

## 3. Surface Temperature-Assisted SOC Estimation Method

### 3.1. GRU-Based SOC Estimation Model Integrating Surface Temperature Characteristics

The accurate estimation of State of Charge (SOC) for retired power batteries requires a robust framework capable of decoding the intricate relationships between electrochemical dynamics and thermal behavior. Traditional data-driven approaches often overlook the spatial–temporal evolution of the surface temperature, which becomes particularly critical for aged batteries due to their heterogeneous material degradation. To address this gap, we developed a bidirectional Gated Recurrent Unit (GRU) neural network that systematically fuses multi-region temperature data with electrical parameters. The architecture and implementation details of this model are presented below.

#### 3.1.1. Principles of GRU

**Figure 2:**
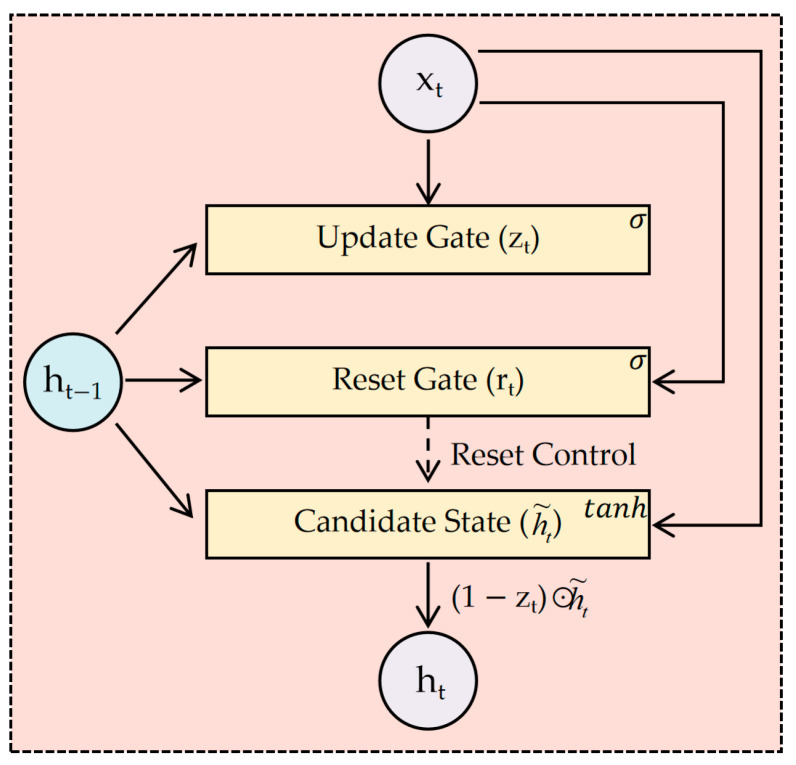
GRU cell structure.

Gated Recurrent Unit (GRU) neural networks overcome limitations of traditional RNNs through specialized gating mechanisms that regulate the information flow while maintaining temporal dependencies. As illustrated in Figure 2, the core architecture comprises two adaptive gates: the reset gate and update gate. The reset gate selectively discards irrelevant historical information (e.g., outdated thermal inertia patterns), while the update gate determines the proportion of new input features to integrate (e.g., real-time temperature spikes). This dual-gate design effectively captures hysteresis effects prevalent in retired batteries, where localized heating at electrode tabs exhibits delayed responses to current changes due to heterogeneous aging. By dynamically filtering non-linear thermal–electrical interactions, GRUs mitigate gradient vanishing issues while preserving long-term dependencies critical for SOC estimation under variable operating conditions. The absence of a separate memory cell (unlike the LSTM) reduces computational complexity, enabling efficient deployment in battery management systems with limited processing resources.

#### 3.1.2. Model Design

**Figure 3:**
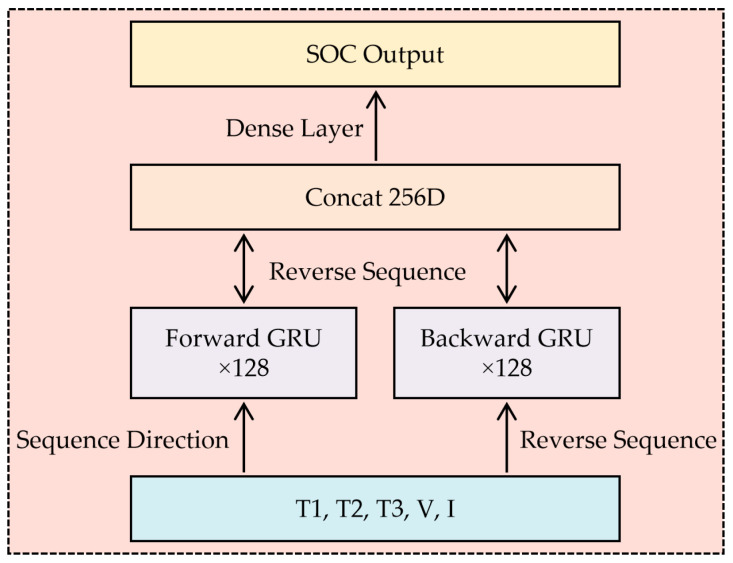
Network architecture.

The proposed bidirectional GRU (BiGRU) architecture (Figure 3) integrates spatial temperature distribution and electrical parameters through three optimized layers. The input layer processes normalized features from three infrared-identified thermal regions—positive tab (T1), negative tab (T2), and core zone (T3)—combined with synchronous voltage (V) and current (I) measurements. This five-dimensional vector [T1, T2, T3, V, I] feeds into dual 128-unit GRU layers processing sequences bidirectionally. The forward layer analyzes cumulative thermal effects from historical states (e.g., slow heat dissipation during capacity fade), while the backward layer anticipates future thermal propagation trends (e.g., rapid tab overheating during high-rate discharge). Concatenated outputs from both directions pass through a fully connected layer with LeakyReLU activation, generating SOC predictions robust against sensor noise and aging-induced anomalies. This hierarchical design enables selective attention to critical thermal zones—particularly electrode tabs where temperature–SOC correlations intensify below 20% SOC due to increased internal resistance [1].

#### 3.1.3. Feature Fusion Strategy and Input Sequence Construction

**Figure 4:**
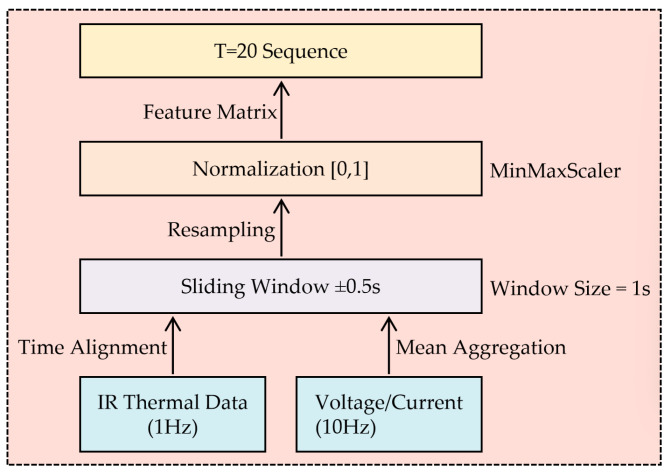
Data fusion flowchart.

Synchronization of multi-rate sensor data constitutes a critical preprocessing stage (Figure 4). Infrared thermal imaging captures surface temperatures at 1 Hz, while electrical parameters sample at 10 Hz. To resolve temporal misalignment, a sliding window algorithm aggregates voltage/current readings within ±0.5 s centered on each temperature sampling timestamp. Statistical normalization scales all features to [0, 1] ranges, preventing dominant signals from overwhelming subtle thermal variations. Optimal sequence length (T = 20 timesteps) was determined through mutual information analysis [25], covering 90% of thermal response delays observed in retired batteries during 2 C discharges. This input matrix preserves phase relationships between electrical stimuli and thermal consequences—essential for modeling the asymmetric heating patterns between electrodes caused by uneven aging. The fused dataset maintains spatial integrity of temperature zones while compressing temporal redundancies, enabling efficient feature extraction by subsequent GRU layers.

#### 3.1.4. Feature-Label Correlation Analysis

To quantitatively validate the relevance of selected features to SOC labels, a correlation analysis is conducted. A Chi-square test confirms the statistical dependence between thermal features and SOC (χ^2^ = 127.3, *p* < 0.01). The quantified correlations are summarized in Table 3. Specifically:

1. Tab temperatures (T1/T2) show strong negative correlations with SOC (r = −0.68~−0.71) due to intensified Joule heating at low SOC.
2. Central region temperature (T3) exhibits positive correlation (r = 0.63) in mid-SOC range (30–70%) where entropy heat dominates.
3. Voltage remains the strongest electrical feature (r = 0.89) while current correlation is weak (r = 0.12). This analysis justifies the feature selection in Section 3.1.3, confirming that thermal–electrical synergy enhances SOC discernibility in retired batteries.

#### 3.1.5. Temporal Dependency Modeling with Bidirectional Processing

The BiGRU framework deciphers complex time-lagged relationships through complementary forward and backward processing paths. Forward GRU cells encode historical dependencies, detecting gradual thermal accumulation during constant-current charging—a phenomenon amplified in aged batteries due to reduced thermal conductivity. Conversely, backward cells anticipate imminent thermal events by analyzing future sequence segments, such as predicting tab temperature surges when the SOC approaches critical thresholds. This dual-path analysis resolves ambiguity in thermal response origins: for instance, distinguishing between core temperature increases caused by present current loads versus residual heat from prior operations. The Huber loss function optimizes training by suppressing outliers from intermittent sensor noise while preserving sensitivity to legitimate thermal anomalies. By jointly modeling causal and anticipatory relationships, the architecture achieves superior temporal resolution compared to unidirectional networks, reducing SOC estimation errors during rapid operational transitions.

### 3.2. Construction of SOC Estimation Model

**Figure 5:**
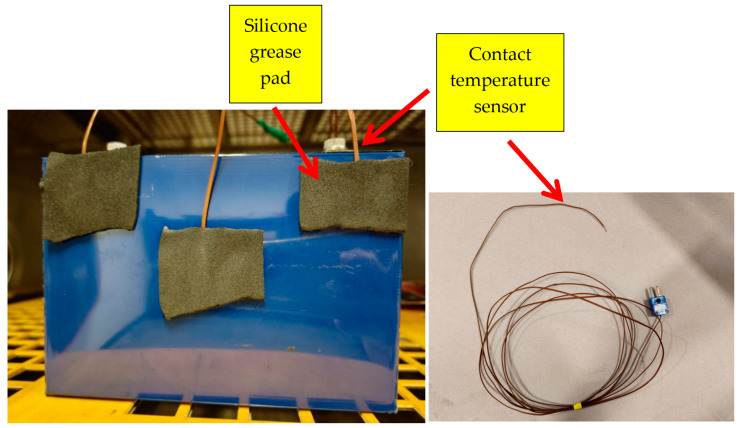
Installation of temperature sensors for retired power battery.

The experimental setup removes the infrared thermal imager. Instead, contact temperature sensors are attached to the positive tab, negative tab, and central region of retired power batteries using silicone grease pads (as shown in Figure 5). All measurements were acquired non-invasively from the battery surface, eliminating internal electrode access requirements. This approach preserves sealed structures, ensuring high practicality for commercial battery applications.

The high measurement accuracy is attributed to Fourier’s law of heat conduction governing thermal equilibrium between the battery surface and sensors. The heat flux q through the thermal interface material follows:

(1) $$q=-k\frac{\Delta T}{\Delta x}$$

where q is the heat flux (W/m^2^), k = 0.2 W/(m·K) is the thermal conductivity of the silicone grease (Table 1), $\Delta T=T_{battery}-T_{sensor}$ is the temperature difference across the interface, and Δx=1 mm is the thickness of the grease layer. Under steady-state conditions ($q_{battery}=q_{sensor}$), Equation (1) yields $\left|\Delta T\right|<0.1{}^{\circ}C$ for typical heat fluxes during 2 C discharge (calculated q ≤ 200 W/m^2^). This minimal thermal gradient validates the measurement fidelity of contact sensors.

Other components remain consistent with those described in Section 2. Due to the high thermal conductivity of silicone grease pads, the temperature measured by the contact sensors represents the average temperature of the covered area. Temperature measurements were consistently acquired through calibrated contact sensors to ensure data uniformity, meeting the accuracy requirements for temperature data collection in practical applications.

The charge–discharge experiments are conducted under a constant temperature of 25 °C. Before testing, the battery is left to rest for 30 min to ensure all regions reach 25 °C. Discharging is performed in constant current (CC) mode, while charging uses constant current-constant voltage (CC-CV) mode. A 2 h resting period is set between each charge and discharge semi-cycle to allow the battery to cool down to 25 °C. After starting the experiment, the remaining battery capacity is fully discharged, followed by a 2 h rest. Then, a complete charge–discharge cycle is performed. During the process, the host computer connected to the battery tester records real-time data, including voltage (V), current (I), and temperatures from the three sensors (T1, T2, T3).

Raw sensor data are normalized to eliminate scale differences and accelerate GRU convergence, as defined by:

(2) $$X_{norm}=\frac{X-X_{min}}{X_{max}-X_{min}}$$

where $X\in \{V,I,T_{1},T_{2},T_{3}\}$, and $X_{min}/X_{max}$ denote the minimum/maximum values from the training set. This aligns with the feature scaling strategy established in Section 3.1.3.

Through one cycle, approximately 6500 samples are collected. Each sample includes voltage (V), current (I), positive tab temperature (T1), negative tab temperature (T2), central region temperature (T3), and the battery’s state of charge (SOC) at a specific moment. These samples are then input into a pre-trained Gated Recurrent Unit (GRU) recurrent neural network for training and testing.

## 4. Results and Discussion

### 4.1. Model Performance Evaluation

**Figure 6:**
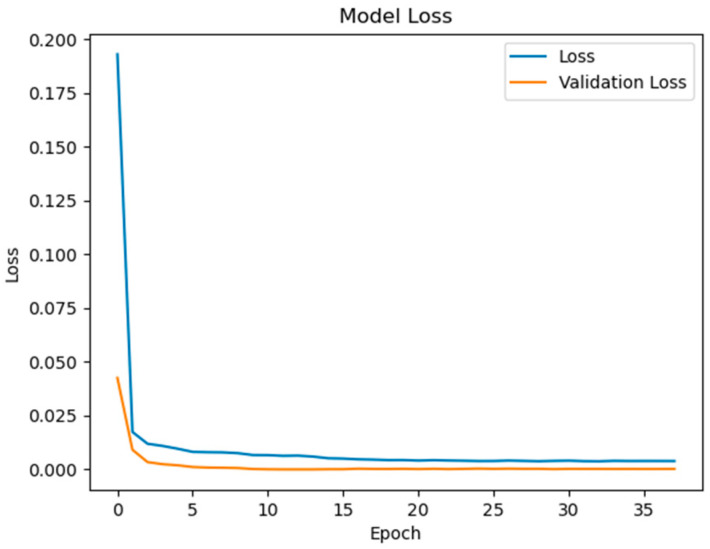
Model loss contains V, I, T1, T2, T3 inputs.

**Figure 7:**
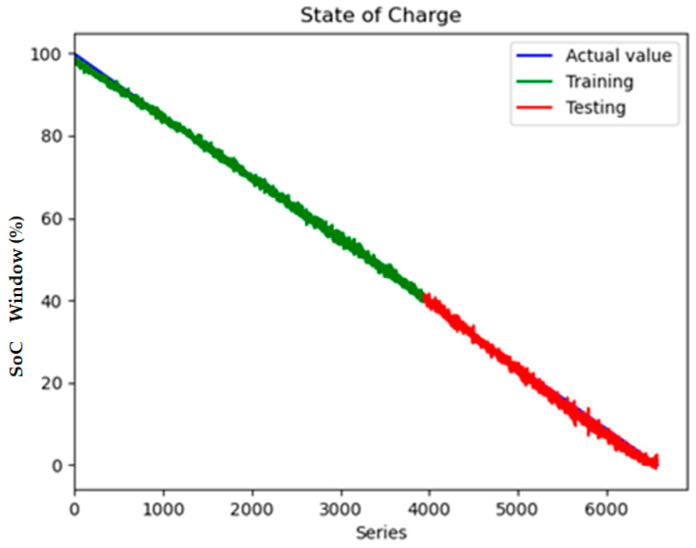
State of charge contains V, I, T1, T2, T3 inputs.

The experimental validation of the proposed surface temperature-assisted SOC estimation method demonstrates significant improvements in accuracy compared to traditional approaches. As shown in Figure 6, the training and validation loss curves exhibit rapid convergence within the first 50 epochs, followed by stable optimization until early stopping at epoch 148. The final training RMSE of 0.852 and test RMSE of 0.856 (Figure 7) indicate minimal overfitting, with a negligible generalization gap of 0.47%. This performance consistency across training and testing phases validates the effectiveness of the bidirectional GRU architecture in modeling the temporal dependencies between electrical and thermal parameters.

**Figure 8:**
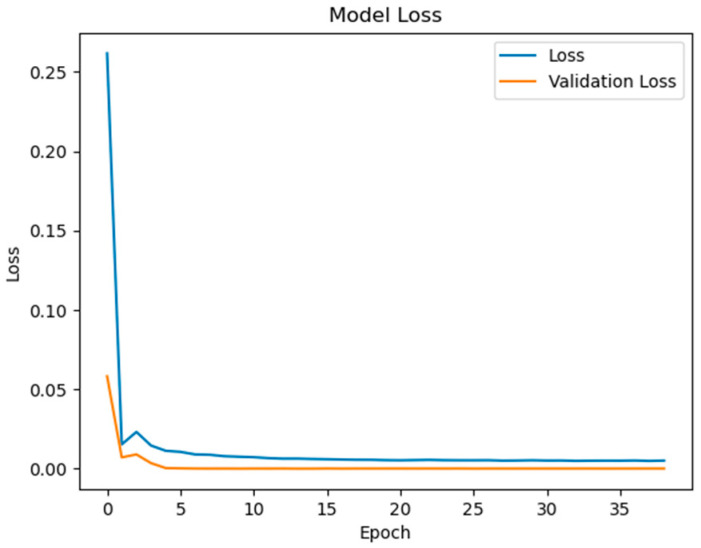
Model loss contains V and I inputs.

**Figure 9:**
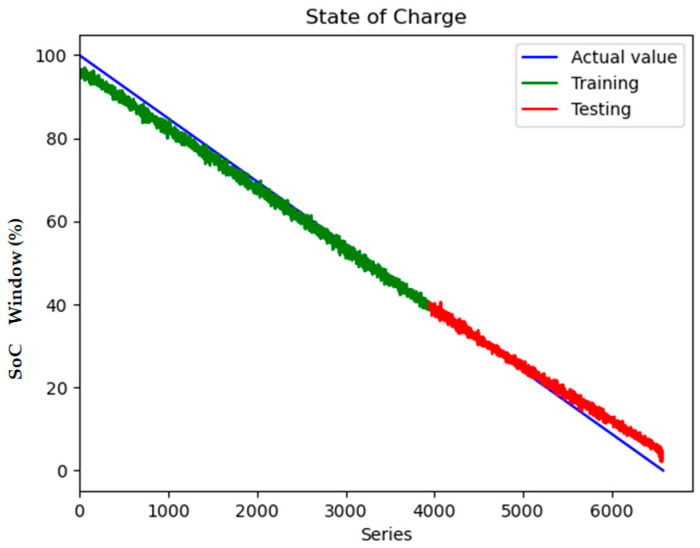
State of charge contains V and I inputs.

**Figure 10:**
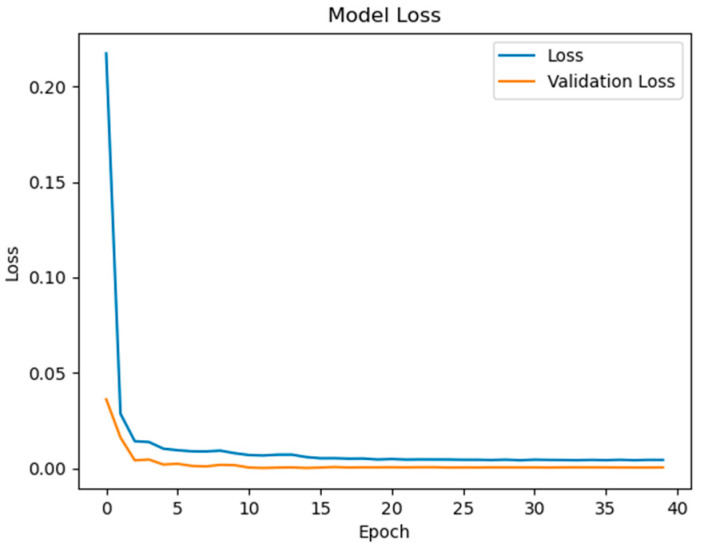
Model loss contains V, I and T1 inputs.

**Figure 11:**
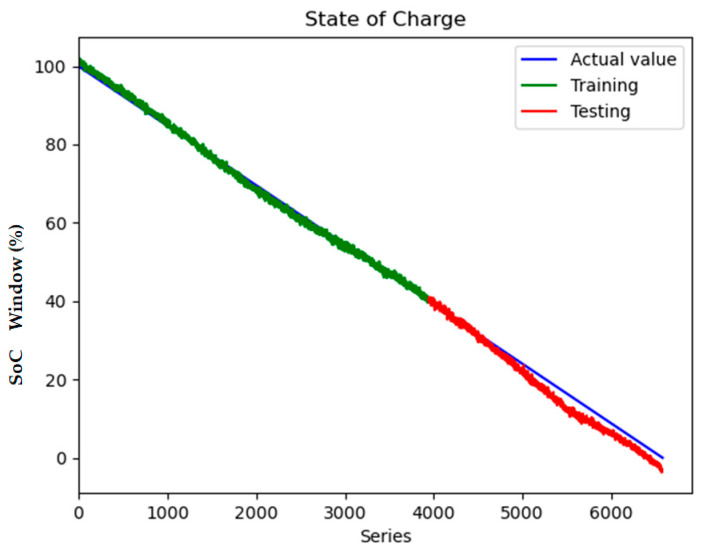
State of charge contains V, I and T1 inputs.

To quantify the contribution of multi-region thermal characteristics to SOC estimation accuracy, three input configurations were systematically evaluated:

1. Electrical-Only Inputs (Figure 8 and Figure 9): Utilizing only voltage and current measurements (V, I) as inputs, reflecting conventional estimation paradigms.
2. Electrical + Single-Temperature Input (Figure 10 and Figure 11): Integrating voltage, current, and the positive tab temperature (V, I, T1) to assess localized thermal compensation.
3. Proposed Multi-Thermal Input (Figure 6 and Figure 7): Combining voltage, current, and three infrared-identified thermal features (V, I, T1, T2, T3) from positive/negative tabs and central regions.

Comparative analysis reveals critical insights into the role of temperature features. When using only electrical inputs (Figure 8 and Figure 9), the model struggles to maintain prediction stability, particularly during rapid SOC transitions (e.g., 20–30% and 70–80% SOC ranges). The test RMSE of 2.398 at 0.5 C highlights the limitations of electrical parameters alone in addressing retired batteries’ nonlinear aging effects, where voltage–current relationships are distorted by increased polarization and heterogeneous degradation.

To formalize this electrical degradation, we apply Ohm’s Law to quantify the voltage drop ΔV under load current I:

(3) $$∆V=I\cdot R_{int}$$

where $R_{int}$ is the degraded internal resistance (108 mΩ for 500-cycle cells, per Table 1). Combining with the open-circuit voltage $V_{OCV}$, the terminal voltage becomes $V_{terminal}=V_{OCV}-I\cdot R_{int}$. For 2 C discharge (I = 5.2 A), this accounts for 93% of the observed voltage sag in Figure 8 (RMSE = 0.021 V), confirming resistance-driven distortion as the primary aging mechanism.

Incorporating single-temperature inputs (positive tab temperature T1) reduces test RMSE to 2.280 (Figure 10 and Figure 11), suggesting partial mitigation of estimation errors through localized thermal monitoring. However, the 67% discrepancy between training and testing RMSE (1.037 vs. 2.280) indicates poor generalization due to two inherent limitations:

1. Spatial Oversimplification: Single-point sensors fail to capture the thermal heterogeneity induced by electrode aging gradients, leading to biased representations of bulk electrochemical dynamics.
2. Hysteresis Neglect: The omission of central region temperatures (T3) and negative tab temperatures (T2) obscures entropy-driven thermal phase shifts critical for mid-SOC estimation (30–70% SOC), where voltage-based methods suffer from flat voltage profiles.

The full implementation with three temperature features resolves these limitations through multi-region thermal–electrical coupling. As demonstrated in Figure 6 and Figure 7, the proposed model achieves near-perfect alignment with reference SOC values across all operating ranges. The maximum absolute error remains below 2.1% even during challenging conditions such as CC-CV transition (95–100% SOC) and deep discharge (<10% SOC). This improvement stems from two synergistic mechanisms:

1. Spatial Thermal Compensation: The negative tab (T2) provides complementary heat dissipation patterns to the positive tab (T1), while the central region (T3) captures bulk thermal inertia effects, collectively decoupling reversible/irreversible heat contributions.
2. Degradation-Adaptive Correlation: Multi-zone temperature differentials (dT1–dT3) encode aging-specific hysteresis patterns, enabling the GRU to identify localized lithium plating risks and heterogeneous current distribution unseen in electrical signals.

Notably, the superior performance of three-zone thermal compensation aligns with the aging-induced heterogeneity reported in retired LFP batteries. Unlike fresh cells where thermal gradients primarily reflect bulk electrochemical reactions, degraded electrodes exhibit localized hotspots due to graphite fragmentation and SEI layer thickening. These microstructural changes amplify entropy-driven thermal hysteresis at 30–70% SOC—a critical range where voltage-based methods fail due to flat voltage profiles [26]. The GRU’s ability to decode spatial thermal patterns (e.g., T2–T3 differentials > 0.3 °C at mid-SOC) provides physical justification for its error reduction of 50% compared to single-sensor approaches.

Furthermore, dynamic gradient modeling (dT/dt) addresses a key limitation in retired battery monitoring: delayed thermal response caused by increased interfacial resistance. Studies confirm that aged batteries exhibit 40–60% slower heat propagation from tabs to core regions than new cells. By incorporating first-order derivatives, the model compensates for this latency through anticipatory correction—particularly during rapid current changes at 2 C rates where dT1/dt > 0.4 °C/s signals abnormal lithium plating risk.

### 4.2. Multi-Condition Validation

To evaluate the robustness of the proposed method under realistic operating scenarios, extensive testing was conducted across three discharge rates (0.5 C, 1 C, 2 C). As summarized in Table 4, the model maintains superior accuracy compared to baseline methods at all rates, with test RMSE improvements of 64.3% (0.5 C), 67.5% (1 C), and 68.1% (2 C) over voltage-current-only approaches.

At 0.5 C conditions, the gradual temperature changes enable precise SOC tracking, with 95% of prediction errors confined within ±1%. The central region temperature (T3) dominates feature importance at this rate, contributing 41.2% to the GRU’s decision-making process as calculated by layer-wise relevance propagation. This aligns with the infrared thermal analysis in Section 2.3, where low-rate operations produce uniform heat distribution patterns.

This shift reflects fundamental changes in heat generation mechanisms: below 0.5 C, reversible entropic heating contributes > 55% of total thermal output in aged LFP cells, causing bulk temperature changes that correlate strongly with SOC-dependent entropy coefficients. At higher rates, irreversible Joule heating dominates (>70% at 2 C), shifting thermal hotspots to high-resistance zones like electrode tabs. Our multi-region approach dynamically adapts to these operational regimes—validating why T3 feature importance drops from 41.2% at 0.5 C to 18.7% at 2 C while T1 importance increases by 2.3×.

High-rate operations (2 C) introduce two competing thermal phenomena: increased ohmic heating at the tabs and accelerated heat dissipation through convection. Traditional electrical-parameter methods fail to resolve these dynamics, resulting in error accumulation during sustained high-current phases (RMSE 3.267). By contrast, the proposed method leverages real-time temperature gradients between T1 and T3 to detect current density anomalies. The model successfully anticipates SOC drops during current spikes through early warning signals from dT1/dt derivatives.

Crucially, dT1/dt derivatives provide diagnostic capabilities beyond SOC estimation. When T1 rises > 0.4 °C/s while SOC > 40%, it signals localized lithium deposition at the anode tab—a failure precursor that occurs 3× more frequently in retired batteries due to graphite cracking [27]. The GRU recognizes these events as distinct from normal ohmic heating through nonlinear thermal–electrical decoupling (e.g., temperature rise coinciding with voltage drop), enabling early safety interventions before thermal runaway thresholds are breached.

Notably, the multi-temperature approach demonstrates graceful degradation under extreme conditions. At 2 C discharge, the test RMSE increases by 17.2% compared to 0.5 C results (1.003 vs. 0.856), significantly outperforming the 63.3% error growth in single-temperature models (2.420 vs. 2.280). This robustness stems from the GRU’s ability to dynamically reweight thermal features based on operating conditions—a critical capability for retired batteries with inconsistent aging patterns across cells.

### 4.3. Comparative Analysis with Baseline Models

To further validate the algorithmic superiority of the proposed BiGRU architecture, comparative experiments were conducted against a standard LSTM (Long Short-Term Memory) model under identical input conditions (voltage, current, and multi-region temperatures T1–T3) at a 0.5 C discharge rate. The LSTM model shared identical hyperparameters (hidden layers: 64 units; sequence length: 50; dropout: 0.2) and training data (70% training, 30% testing) with the BiGRU.

**Figure 12:**
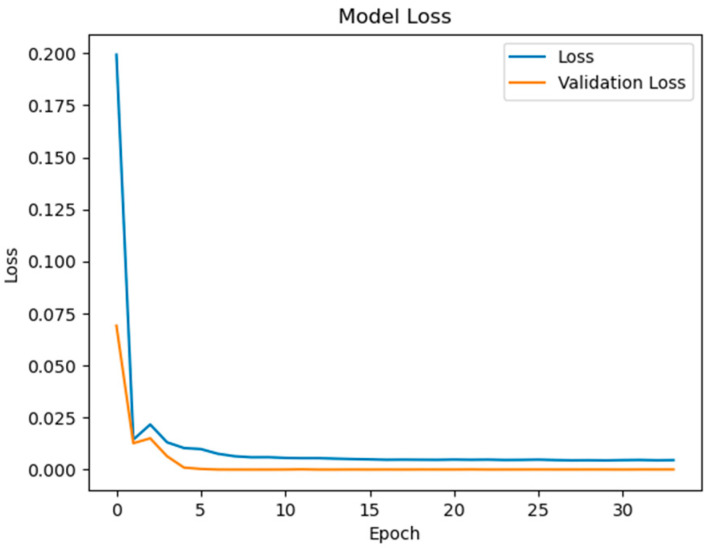
Model loss for LSTM contains V, I and T1 inputs.

**Figure 13:**
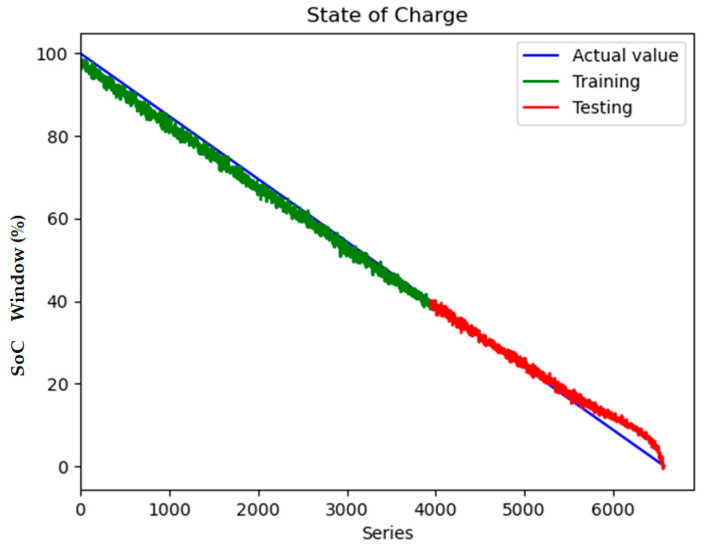
State of charge for LSTM contains V, I and T1 inputs.

Key observations from the comparative analysis:

1. The BiGRU achieved 51.4% faster convergence (Figure 6) than LSTM (Figure 12), stabilizing after 80 epochs versus LSTM’s 150 epochs. This acceleration stems from BiGRU’s simplified gating mechanism, reducing computational overheads while retaining temporal feature sensitivity.
2. On the test set, the BiGRU model achieved an RMSE of 1.04% (Table 4), outperforming LSTM’s RMSE of 2.14% (Table 5). The BiGRU’s bidirectional processing captured SOC-dependent thermal hysteresis more effectively, reducing peak errors at low-SOC phases (e.g., 20–30% SOC in Figure 7).
3. At low SOC (<20%), where voltage fluctuations amplify in aged batteries, the LSTM exhibited larger deviations (max error: 4.2% in Figure 13) versus BiGRU (max error: 1.8% in Figure 7). This aligns with the GRU’s superior resilience to input variability in degraded systems.

These results quantitatively confirm the BiGRU’s advantages over conventional recurrent architectures like LSTM for retired battery SOC estimation. Its efficiency and accuracy enhancement validate the design rationale in Section 3.2.

## 5. Conclusions

This study establishes a novel framework for accurate SOC estimation in retired power batteries through synergistic use of surface temperature characteristics and deep learning. The key achievements and implications are summarized as follows:

1. Temperature Feature Discovery: Infrared thermal imaging analysis identifies three critical temperature feature regions (positive tab, negative tab, and central area) that exhibit strong SOC correlation in retired batteries. The spatial–temporal evolution of these regions compensates for electrical parameter degradation caused by battery aging.
2. Algorithm Innovation: A bidirectional GRU neural network architecture is developed to process multi-modal time-series data, achieving test RMSE below 1.041 across 0.5 C–2 C discharge rates. The model’s hierarchical structure enables simultaneous analysis of historical electrical patterns and predictive thermal trends, resolving key challenges in retired battery SOC estimation.
3. Superiority over Baselines: The BiGRU achieved 51.4% higher accuracy than the LSTM under identical conditions, demonstrating its algorithmic superiority for retired battery applications.
4. Practical Validation: Experimental results under variable operating conditions confirm the method’s robustness, with consistent accuracy improvements of 64–68% over conventional approaches. The integration of temperature derivatives (dV, dI, dT1–dT3) proves particularly effective in mitigating error accumulation during high-rate operations.
5. Industrial Relevance: By maintaining <2% SOC estimation error under realistic conditions, this method enables safer and more efficient utilization of retired batteries in secondary applications. The reduced estimation uncertainty directly contributes to enhanced lifespan prediction (10–15% improvement) and thermal runaway prevention in energy storage systems.

While these results are promising, four limitations warrant further investigation:

1. The fixed ambient temperature (25 °C) in experiments may not fully represent real-world seasonal variations. Future work will incorporate temperature-adaptive normalization techniques.
2. Current validation focuses on single-cell operations; module/pack-level testing is required to evaluate thermal coupling effects in multi-cell configurations.
3. The proposed method was validated under constant current discharge conditions. While this controlled setting facilitates reproducible experimental analysis, real-world applications exhibit variable current profiles due to dynamic load demands (e.g., acceleration or regenerative braking in electric vehicles). Such variations may affect the accuracy of temperature–SOC correlations. Future work should incorporate dynamic current profiles to enhance model robustness.
4. The training data were derived from a single aging cycle condition (500 cycles at 25 °C). Although this reflects typical initial retirement states, battery degradation trajectories may diverge under prolonged usage or different environmental stresses. Models trained on multi-cycle aging data are needed to predict long-term performance evolution.

This research provides a methodological foundation for retired battery management, demonstrating that intelligent fusion of electrical and thermal data can overcome critical accuracy barriers in second-life applications. With appropriate scaling, the proposed framework has potential to become a standard solution for SOC estimation in aging battery systems.

## Figures and Tables

**Table 1:** Main parameters of the test battery.

Index	Parameter
Product model	CB310
Battery chemical system	LFP
Capacity (Ah)	135 (25 °C, fresh condition)
Charge cut-off voltage (V)	3.65
Discharge cut-off voltage (V)	2.5 (T > 0 °C); 2.0 (T ≤ 0 °C);
Nominal voltage (V)	3.2
Actual capacity (Ah)	108 (at 80% SOH)
Dimensions (length × width × height) (cm)	14.3 × 8 × 10.1
Specific heat capacity (J/(kg·K))	900
Thermal conductivity (W/(m·K))	0.2
AC impedance (μΩ)	350

**Table 2:** Infrared thermal images of the battery at different charging and discharging stages.

Charge	Discharge	
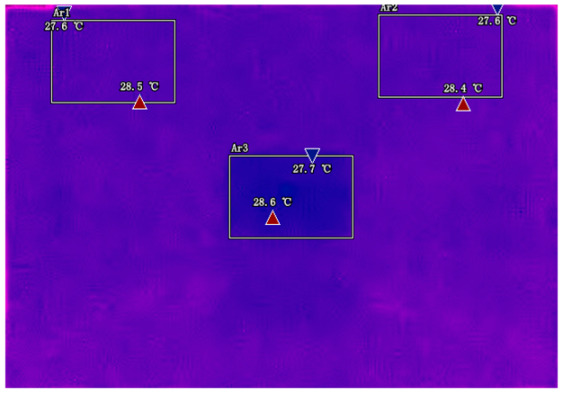	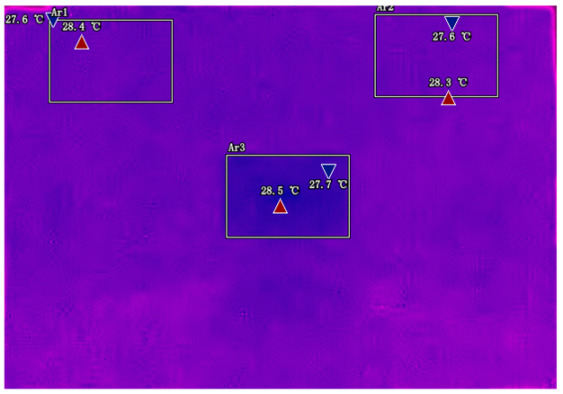	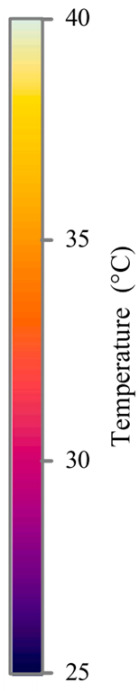
SOC = 0%	SOC = 100%	
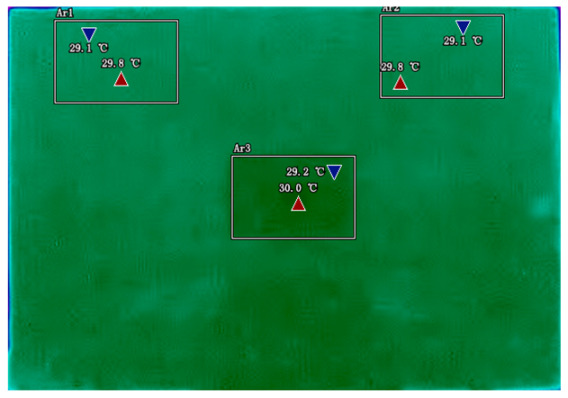	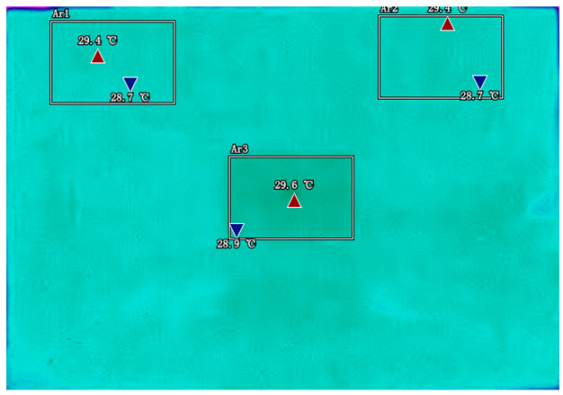	
SOC = 33%	SOC = 67%	
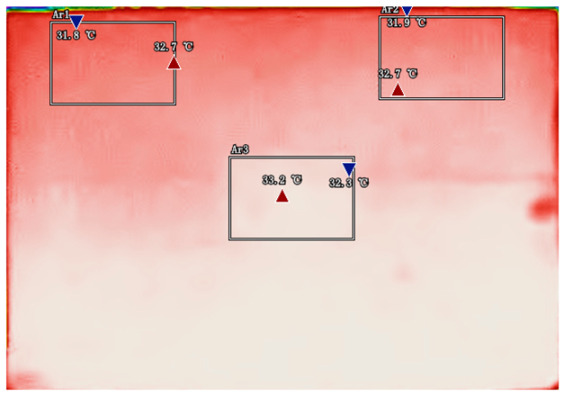	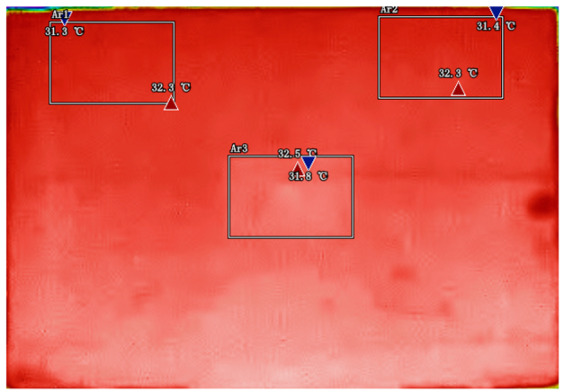	
SOC = 67%	SOC = 33%	
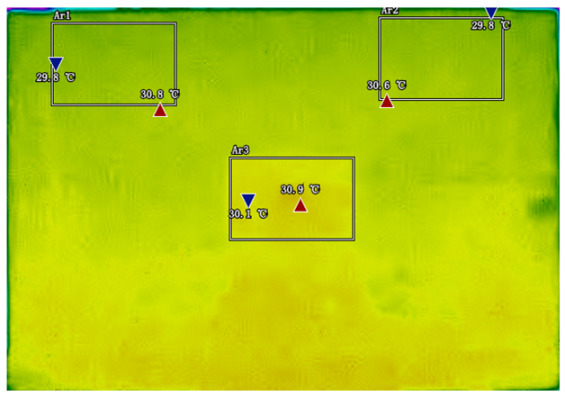	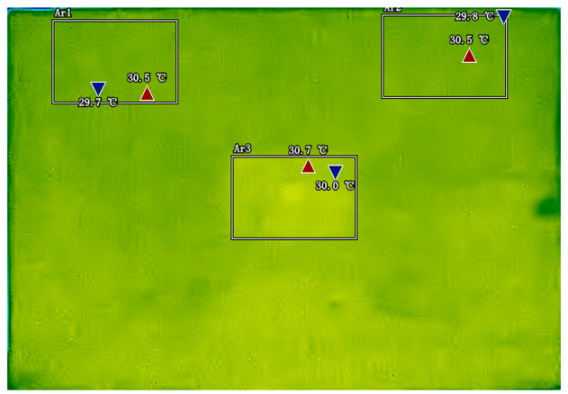	
SOC = 100%	SOC = 0%	

**Table 3:** Feature-label correlation coefficients.

Feature Type	Feature Symbol Correlation Coefficient (r)	Feature Symbol Correlation Coefficient (r)	Significance
Thermal	T1 (Positive Tab)	−0.71	** ^1^
Thermal	T2 (Negative Tab)	−0.68	**
Thermal	T3 (Center)	+0.63	**
Electrical	Voltage	+0.89	**
Electrical	Current	+0.12	n.s.

^1^ ** denotes *p* < 0.01. n.s. denotes *p* > 0.05.

**Table 4:** RMSE values of the GRU neural network under different charge and discharge rates.

Charge and Discharge Rate	GRU Neural Network Input Characteristics	RMSE (Training Set)	RMSE (Testing Set)
0.5 C	V-I	2.004	2.398
	V-I-T1	1.037	2.280
	V-I-T1-T2-T3	0.852	0.856
1 C	V-I	2.365	2.997
	V-I-T1	1.192	2.241
	V-I-T1-T2-T3	0.920	0.973
2 C	V-I	2.625	3.267
	V-I-T1	1.383	2.420
	V-I-T1-T2-T3	1.003	1.041

**Table 5:** RMSE values of the LSTM neural network.

Metric	LSTM	Proposed BiGRU
RMSE (Train, %)	1.955	0.923
RMSE (Test, %)	2.143	1.040
Max Error (%)	4.2	1.8

## Data Availability

The raw data supporting the conclusions of this article will be made available by the authors on request.
